# 2083. Waning of Bivalent mRNA Vaccine Effectiveness Against COVID-19-associated Hospitalization Among Immunocompetent Adults Aged ≥65 Years – IVY Network, 20 U.S. States, September 8, 2022-April 1, 2023

**DOI:** 10.1093/ofid/ofad500.153

**Published:** 2023-11-27

**Authors:** Jennifer DeCuir, Yuwei Zhu, Manjusha Gaglani, Adit A Ginde, Nicholas Mohr, Kevin Gibbs, David Hager, Anne Frosch, Amira Mohamed, Nicholas Johnson, Jay S Steingrub, Ithan Peltan, Emily T Martin, William Bender, Jennifer Wilson, Nida Qadir, Christopher Mallow, Jennie H Kwon, Matthew Exline, Adam S Lauring, Cristie Columbus, Ivana Vaughn, Basmah Safdar, James Chappell, Adrienne Baughman, Kelsey N Womack, Sydney A Swan, Meredith L McMorrow, Wesley Self, Diya Surie

**Affiliations:** Centers for Disease Control and Prevention, Atlanta, GA; Vanderbilt University, Nashville, Tennessee; Baylor Scott & White Health, Temple, TX; University of Colorado, Aurora, Colorado; University of Iowa, Iowa City, Iowa; Wake Forest University Baptist Medical Center, Winston-Salem, North Carolina; Johns Hopkins Hospital, Baltimore, Maryland; Hennepin County Medical Center, Minneapolis, Minnesota; Montefiore medical center, Bronx, New York; University of Washington School of Medicine, Seattle, Washington; Baystate Medical Center, Springfield, Massachusetts; Intermountain Medical Center and University of Utah, Salt Lake City, Utah; University of Michigan, Ann Arbor, MI; Emory University School of Medicine, Atlanta, Georgia; Stanford University School of Medicine, Stanford, California; Ronald Reagan UCLA Medical Center, Los Angeles, California; University of Miami, Miami, Florida; Washington University - School of Medicine, St. Louis, MO; Ohio State University Wexner Medical Center, Columbus, Ohio; University of Michigan, Ann Arbor, MI; Baylor Scott & White Health - Dallas, Dallas, Texas; Henry Ford Health, Detroit, Michigan; Yale University School of Medicine, New Haven, Connecticut; Vanderbilt University Medical Center, Nashville, Tennessee; Vanderbilt University Medical Center, Nashville, Tennessee; Vanderbilt University Medical Center, Nashville, Tennessee; Vanderbilt University Medical Center, Nashville, Tennessee; CDC/NCIRD/CORVD/SPB, Atlanta, GA; Vanderbilt University Medical Center, Nashville, Tennessee; Centers for Disease Control and Prevention, Atlanta, GA

## Abstract

**Background:**

On September 1, 2022, the Advisory Committee on Immunization Practices recommended a bivalent mRNA COVID-19 booster dose for persons who had completed at least a primary COVID-19 vaccination series ≥2 months earlier. Early data showed high effectiveness of a bivalent booster in preventing COVID-19-associated hospitalization within 45 days of receipt; however, little is known about the durability of this protection.

**Methods:**

Data from the Investigating Respiratory Viruses in the Acutely Ill (IVY) Network were used to conduct a case-control analysis measuring bivalent vaccine effectiveness (VE) against COVID-19–associated hospitalization over time. During September 8, 2022–April 1, 2023, immunocompetent, hospitalized adults aged ≥65 years with COVID-19-like illness were enrolled at 25 hospitals in 20 U.S. states. COVID-19 case-patients tested positive for SARS-CoV-2 by a nucleic acid or antigen test within 10 days of illness onset, while control-patients tested negative for SARS-CoV-2 during the same interval. Multivariable logistic regression was used to measure absolute and relative bivalent VE adjusted for age, sex, race and ethnicity, admission date, and U.S. Health and Human Services region. Unvaccinated patients and patients who received 2–4 doses of monovalent-only mRNA vaccine were used as the reference group for absolute and relative VE, respectively. Bivalent VE was calculated for 7–89 days and 90–179 days from booster dose receipt to illness onset.

**Results:**

A total of 2,787 immunocompetent, hospitalized adults aged ≥65 years were enrolled in the IVY Network during the study period (1,236 COVID-19 case-patients and 1,551 control patients). Absolute VE of a bivalent booster dose against COVID-19-associated hospitalization was 58% (95% CI=42%–70%) after 7–89 days and 27% (95% CI= -7% to 50%) after 90–179 days. Relative VE of a bivalent booster dose was 54% (95% CI=41%–64%) after 7–89 days and 19% (95% CI= -8% to 39%) after 90–179 days (Figure).

**Figure:**
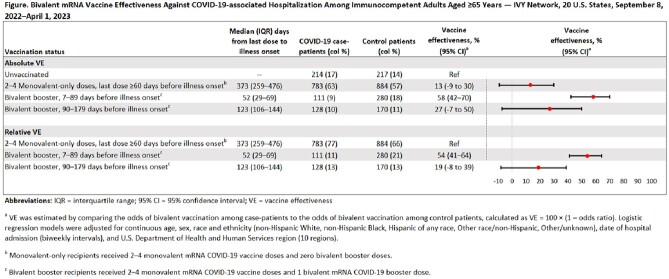

**Conclusion:**

Bivalent mRNA vaccination provided moderate protection against COVID-19-associated hospitalization within 90 days of receipt among adults aged ≥65 years, with waning protection after 90 days. Additional booster doses could improve protection against COVID-19-associated hospitalization among older adults.

**Disclosures:**

**Adit A. Ginde, MD, MPH**, AbbVie: Grant/Research Support|Faron Pharmaceuticals: Grant/Research Support **Ithan Peltan, MD**, Asahi Kasei Pharma: Institutional support|Regeneron: Institutional support **Emily T. Martin, PhD, MPH**, Merck: Grant/Research Support **Matthew Exline, MD**, Abbott Labs: Honoraria|Regeneron: Grant/Research Support **Adam S. Lauring, MD, PhD**, Roche: Advisor/Consultant|Sanofi: Advisor/Consultant

